# The myriad roles of RNA structure in the flavivirus life cycle

**DOI:** 10.1080/15476286.2024.2357857

**Published:** 2024-05-26

**Authors:** Quinn H. Abram, Breanna N. Landry, Alex B. Wang, Ronja F. Kothe, Hannah C.H. Hauch, Selena M. Sagan

**Affiliations:** a Department of Biochemistry, McGill University, Montreal, QC, Canada; b Department of Microbiology & Immunology, University of British Columbia, Vancouver, BC, Canada; c Department of Microbiology & Immunology, McGill University, Montreal, QC, Canada

**Keywords:** Flavivirus, Zika virus, dengue virus, translation, replication, packaging, stem-loop A, RNA-dependent RNA polymerase, methyltransferase, replication organelle

## Abstract

As positive-sense RNA viruses, the genomes of flaviviruses serve as the template for all stages of the viral life cycle, including translation, replication, and infectious particle production. Yet, they encode just 10 proteins, suggesting that the structure and dynamics of the viral RNA itself helps shepherd the viral genome through these stages. Herein, we highlight advances in our understanding of flavivirus RNA structural elements through the lens of their impact on the viral life cycle. We highlight how RNA structures impact translation, the switch from translation to replication, negative- and positive-strand RNA synthesis, and virion assembly. Consequently, we describe three major themes regarding the roles of RNA structure in flavivirus infections: 1) providing a layer of specificity; 2) increasing the functional capacity; and 3) providing a mechanism to support genome compaction. While the interactions described herein are specific to flaviviruses, these themes appear to extend more broadly across RNA viruses.

## Introduction

The *Flavivirus* genus includes several emerging and re-emerging viral pathogens of public health concern. The most well-known of these are the mosquito-borne flaviviruses, which include Zika virus (ZIKV), dengue virus (DENV), West Nile virus (WNV), yellow fever virus (YFV) and Japanese encephalitis virus (JEV); however, this genus also includes tick-borne, insect-specific, and no-known vector flaviviruses [1]. Billions of people around the globe remain at risk of flavivirus infections, with climate change increasing the geographic risk as the range of their insect vectors expands [2,3]. Despite their high-risk profile, we still lack effective vaccines and/or antiviral treatments for many flaviviruses, necessitating a better understanding of the intricacies of their life cycles.

**Figure 1.**
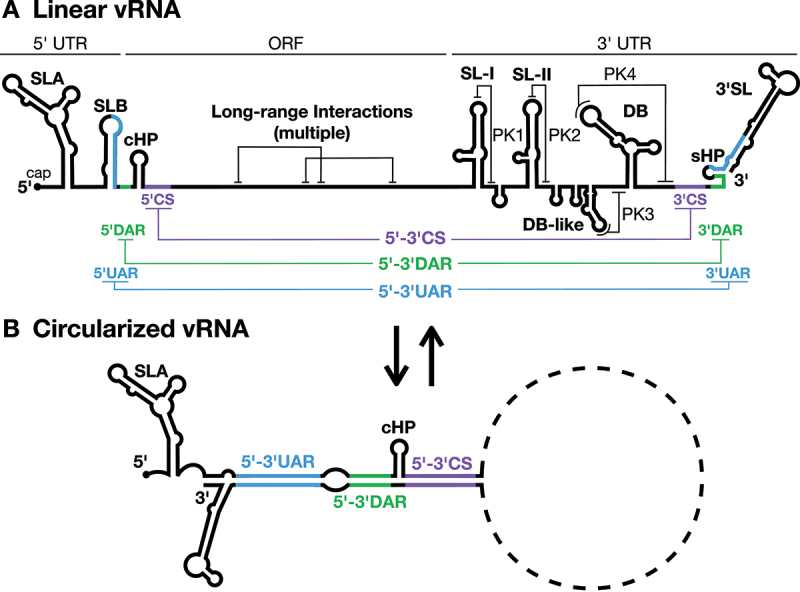
Flavivirus genome organization.

Flaviviruses enter cells through receptor-mediated endocytosis and subsequent endosome acidification triggers viral uncoating, releasing the viral genomic RNA into the cytoplasm. The capped ~11 kb flavivirus genome contains a single open reading frame that encodes a ~ 3400 amino acid polyprotein (Figure 1). Upon uncoating, the positive-sense genomic RNA is immediately able to recruit the ribosome to produce the viral polyprotein, which is processed by both cellular and viral proteases into the 10 mature viral proteins. This includes three structural proteins (capsid, envelope, and pre-membrane) that form the viral particle, and seven non-structural (NS) proteins (NS1, NS2A, NS2B, NS3, NS4A, NS4B and NS5), which coordinate viral replication, virion assembly, and evasion of cellular antiviral responses. Once sufficient translation of the viral proteins has occurred, the genome is condensed into virally induced invaginations of the endoplasmic reticulum (ER) membrane to form the replication organelle (RO), the site of viral genome replication [4,5]. Within the RO, genome replication occurs via synthesis of a negative-strand replicative intermediate, followed by subsequent positive-strand genomic RNA synthesis. Together, the NS3 protein, which has protease, helicase, and nucleotide triphosphatase activities, and the NS5 protein, which has RNA-dependent RNA polymerase (RdRp) and methyltransferase (MTase) activities, contain all the enzymatic activity required for viral RNA replication and capping of newly synthesized positive-sense RNAs. Following genome replication, newly synthesized positive-sense genomic RNAs are released from the RO, where they can either undergo translation and eventually seed a new RO, or can be transported to the assembly site to be packaged into progeny virions [4,5]. Viral particles are assembled on the ER membrane adjacent to ROs and bud into the ER before exiting the cell via the secretory pathway.

The viral RNA genome (vRNA) of flaviviruses has a single open-reading frame (ORF) flanked by highly structured 5´ and 3´ UTRs that possess *cis*-acting RNA structural elements required for viral translation, RNA replication, and assembly. The Zika virus (ZIKV) genome is depicted. A) In the linear (translation-competent) form of the vRNA, the capped viral genomic RNA contains stem-loop A (SLA) and SLB in the 5´ untranslated region (UTR), which lie just upstream of the capsid coding-region hairpin (cHP). The 3´ UTR contains a variable region, with one or more stem-loop structures (SL-I and SL-II), and a dumbbell (DB) region, containing one or more DB sequences, all of which can form local pseudoknot (PK) structures. These structures are followed by the short hairpin (sHP) and the 3´ stem loop (3´SL) at the 3´ terminus of the genome. B) In the circular (replication-competent) form of the viral genome, long-range RNA-RNA interactions between elements in the 5´ and 3´ UTRs (5´-3´CS, 5´-3´DAR, 5´-3´UAR) facilitate cyclization of the vRNA. These long-range interactions partially disrupt the 3´SL structure, and fully disrupt the SLB and sHP structures. UAR, upstream of AUG region; DAR, downstream of AUG region; CS, cyclization sequence.

Notably, the viral positive-sense genomic RNA must serve as a template for all the intracellular steps of the viral life cycle, including translation, replication, and packaging. As such, the genomic RNA itself must be highly dynamic and readily able to accommodate unwinding, elongation, and exposure of different regions of the RNA to viral and cellular proteins in a temporally and spatially regulated manner. Recent innovations in RNA structure-probing techniques have provided novel insights into the RNA structures involved in the flavivirus life cycle and their dynamics (*see* **Box 1**). In this review, we first provide an overview of flavivirus *cis*-acting RNA elements and then highlight the roles of these RNA structural elements and their interplay in the various stages of the viral life cycle. In doing so, we emphasize recent advances that have reshaped our understanding of flavivirus translation, the switch from translation to replication, negative- and positive-strand RNA synthesis, and virion assembly.

**Table:** 

**Box 1. How is RNA structure determined experimentally?** Traditionally, RNA structure has been analysed via indirect methods that modify unpaired and/or unbound ribonucleotides. For example, Selective 2´ Hydroxyl Acylation analysed by Primer Extension (SHAPE) reagents preferentially react with the 2´OH group of unconstrained ribonucleotides to form 2´-*O*-adducts, while dimethyl sulfate (DMS) reacts with specific N atoms in the rings of unpaired adenines and cytidines [6–8]. In both cases, these modifications can be read as terminations of reverse transcription (RT) on the treated RNA. Coupled with high-throughput sequencing, it is now possible to apply these methods on a genome-wide scale. To increase robustness and permit data collection from RNAs with multiple modifications, these approaches utilize mutational profiling (MaP), an approach where the RT conditions are altered such that it introduces a mutation at the modification site on the RNA instead of terminating the reaction [9,10]. As a result, each modification results in a base change that can be detected during sequencing. Advances in sequencing technologies have also resulted in the proliferation of multiple direct methods to assess genome-wide RNA structure, such as psoralen analysis of RNA interactions and structures (PARIS) and cross-linking of matched RNAs and deep sequencing (COMRADES) [11,12]. These more direct methods utilize chemical probes that crosslink base-paired ribonucleotides followed by sequencing of the crosslinked pieces of RNA. However, both the indirect and direct approaches suffer from a shared limitation in that the resulting structural data is an average of all RNA conformations present during treatment. Excitingly, recent advances in the bioinformatic pipelines to analyse this structural data, like DRACO, a tool that deconvolutes different RNA conformations from MaP datasets, may aid in dissecting the heterogeneity of the RNA structurome [13]. Finally, the most direct approaches to assess RNA structure are methods such as cryo-electron microscopy and x-ray crystallography, which have recently been applied in the analysis of the flavivirus SLA structure [14,15]. However, identifying conditions to analyse RNA structures via these methods is not trivial, and at present is largely limited to isolated local structures. Furthermore, additional structure-stabilizing sequences or proteins are often required to sufficiently stabilize the RNA of interest, selectively constraining the RNA in a particular conformation, which may or may not be a biologically relevant conformation [14,15]. As such, combinations of RNA structure-probing techniques (more thoroughly reviewed in [16]), are likely necessary to further our understanding of flavivirus RNA structures.

## Organization of cis-acting RNA elements in the flavivirus genome

Flavivirus genomic RNAs are known to be highly structured, with the 5´ and 3´ ends of the genome being particularly well characterized (reviewed in [17]) (Figure 1 and Table 1). Briefly, the 5´ untranslated region (UTR) contains stem-loop A (SLA) and SLB which are essential for viral RNA replication [18,19]. SLB also contains the 5´ upstream of AUG region (5´UAR), a sequence that is essential for a long-range RNA-RNA interaction that aids in genome cyclization and viral replication [20]. Within the coding sequence, the stable capsid coding-region hairpin (cHP, Figure 1) is required for efficient translation and viral RNA replication [21,22]. Adjacent to cHP lies the 5´ downstream of AUG region (5´DAR) and 5´ cyclization sequence (5´CS), additional sequences that aid in genome cyclization for viral RNA replication (Figure 1) [20,23].

**Table 1.** RNA structures in the flavivirus genome and their roles in the viral life cycle.

Structure		Location in Genome	Interactor(s)	Role(s) in Flavivirus Life Cycle
SLA		5´ UTR	NS5	Translational shut-off Negative-strand RNA synthesis Capping *RO biogenesis?* *Positive-strand RNA synthesis?*
SLB		5´ UTR	NS5	*Translational shut-off?*
cHP		Capsid coding region	Ribosomes	Start site selection
SL-I		3´ UTR	XRN-1	Generation of sfRNAs (immune evasion)
SL-II				
DB-I				
DB-II (or DB-like)				
3´ UTR	SL-II (ZIKV)	3´ UTR	NS2A	Packaging (specificity)
	DB-I to 3´SL (DENV)			
3´SL		3´ UTR	NS5	Negative-strand RNA synthesis *Translational shut-off?*
Cyclization elements	UAR	5´ and 3´ UTRs	Partner cyclization element	Translational shut-off Genome cyclization Negative-strand RNA synthesis
	DAR			
	CS			
Long-range RNA-RNA interactions (>0.5 kb)		Multiple throughout genome	Capsid	Packaging (genome compaction) *RO biogenesis?*
SLA´		3´ terminus of negative-strand	*NS5?*	*Positive-strand RNA synthesis?*

Specific RNA structures in the 3´ UTR also play roles in viral translation, RNA replication, and virion assembly. The 3´ UTR of mosquito-borne flaviviruses can be divided into three distinct domains (Figure 1). The first consists of the variable region, which typically contains one or more stem-loop (SL) structures; while the second consists of one to two dumbbell (DB) structures [24]. Interestingly, both the SL and DB structures form local pseudoknots (PKs) that are 5´-3´ exoribonuclease 1 (XRN-1)-resistant, resulting in the generation of subgenomic flavivirus RNAs (sfRNAs). These sfRNAs have been implicated in inhibition of cellular antiviral responses and in host adaptation between mammals and their mosquito vectors [25]. The final domain contains the 3´CS (which forms a long-range RNA-RNA interaction with the 5´CS), short hairpin (sHP), and the 3´SL [20,26]. The sHP and 3´SL are required for viral RNA replication and contain the 3´UAR and 3´DAR elements that help mediate genome cyclization with the 5´ end of the viral RNA [24,26,27]. Notably, the 3´ UTR has also been implicated in viral genome packaging through interactions with the viral NS2A protein, a small hydrophobic protein implicated in both RO biogenesis and virion assembly [28,29].

In addition to these well-characterized structures in the 5´ and 3´ UTRs, whole genome chemical mapping and cross-linking approaches have demonstrated a high degree of local and long-range secondary and tertiary structure across the flavivirus genome and a dynamic interplay between these structures [30–33]. While there is still much left to learn regarding these RNA structures and their dynamics, the body of knowledge regarding the myriad roles of RNA structure in the viral life cycle continues to grow and inform our understanding of the complex and tightly regulated life cycles of flaviviruses. In the following sections, we provide a summary of recent advances that have shaped our understanding of how the viral RNA itself contributes to the coordination of viral translation, negative- and positive-strand RNA synthesis, RNA capping, and genome packaging. In light of these advances, we describe three major themes regarding the roles of RNA structure in the flavivirus life cycle: 1) RNA structure often provides specificity to the tightly regulated viral life cycle; 2) the ability of the RNA to fold into complex three-dimensional structures increases the functional capacity of the RNA beyond its sequence alone; and 3) the ability to form long-range RNA-RNA interactions likely facilitates viral genome compaction.

## How does the flavivirus genomic RNA direct and regulate viral polyprotein production?

Flavivirus genomes contain a canonical type I (m^7^GpppN) cap structure and are thus primarily translated via cap-dependent translation, utilizing the same mechanism as cellular mRNAs. However, in most mosquito-borne flaviviruses, the start codon is in a canonically poor translation initiation context, often 40–50 nucleotides (nt) upstream of another start codon in a more favourable context [22]. Despite this, initiation from the first start codon is heavily favoured in both mammalian and mosquito cells [22,34,35].

This start codon preference is driven by the cHP, a small stem-loop structure that occurs approximately 12–16 nt downstream of the start codon (Figure 1 and Table 1) [22]. Selection of the correct start codon occurs in a structure-dependent but sequence-independent manner, in which the positioning of the structure relative to the start codon appears to be the critical factor [22,33,36]. Ribosomal pausing at the cHP structure thus appears to help select for the proper start codon, providing specificity to the translation initiation process [22]. Despite this, flavivirus translation efficiency is not substantially affected by defects in cHP structure that result in improper start codon selection, raising the question of why proper start codon selection is maintained across flaviviruses [21,22]. One possible explanation is that proper start codon selection helps ensure the production of the full-length capsid protein. Without the cHP, initiation of translation in DENV occurs at a slightly downstream start codon resulting in loss of the first 15 amino acids of the capsid protein [22,37]. Interestingly, this region contains one of the two basic amino acid clusters at the N-terminus of the capsid protein that are critical for infectious particle production in DENV, and presumably interact with the viral RNA genome during the encapsidation process [37]. However, it is still unclear whether this region is strictly required for packaging across all flaviviruses, as YFV can tolerate N-terminal capsid protein deletions of up to 40 amino acids while maintaining infectious particle production [38]. Nonetheless, it is possible that YFV is the outlier in this regard, considering the strict conservation of the cHP structure across the *Flavivirus* genus, and the need for a sufficient level of positive-charge in the disordered N-terminus of the viral capsid protein for infectious particle production [22,37,38].

In addition to the cHP, the viral 3´ UTR has also been shown to act as a translational enhancer, although the precise mechanism remains unclear [39–42]. Notably, while capping of viral genomic RNAs is critical for successful infection, uncapped genomic RNAs are also capable of establishing productive infections when introduced into susceptible and permissive cells (*see* **Box 2**) [39–43]. Thus, in addition to the cap structure, *cis*-acting RNA elements in the genomic RNA, including the 5´ UTR, cHP, and 3´ UTR, all contribute to translational regulation, thereby increasing the overall functional capacity of the flavivirus genome.

**Table:** 

**Box 2. Can flaviviral RNAs be translated via both cap-dependent and cap-independent mechanisms?** In addition to canonical cap-dependent translation, several flaviviral RNAs have been demonstrated to be capable of cap-independent translation in both mammalian and mosquito cells [39–41]. However, translation of uncapped flaviviral RNAs is much less efficient, with approximately 100-fold lower translation than their capped flaviviral RNA counterparts, and the precise mechanism for this noncanonical translation is still unclear [39]. There remains debate whether the 5´ UTR of flaviviruses has internal ribosome entry site (IRES) activity, with the 5´ UTRs of both DENV and ZIKV passing the classical IRES test of being capable of driving translation of an internal reporter in a bicistronic reporter construct [42,43]. However, inclusion of the 5´ UTR alone resulted in significant, albeit weak, cap-independent translation in these studies [42,43]. Moreover, cap-independent activity was negligible in monocistronic reporter RNAs when the canonical m^7^GpppG cap was replaced with an ApppA cap structure that cannot interact with the eIF4E cap-binding protein and recruit the ribosome [42]. In general, the ability of uncapped reporter RNAs to be translated appears to be dependent upon both the 5´ and 3´ UTRs of the flaviviral genomic RNA, with the 3´ UTR acting as some kind of translational enhancer [39–42]. Nevertheless, it remains unclear which circumstances would necessitate a requirement for such cap-independent translation. One possible explanation could be that cap-independent translation may provide flaviviruses with a counter defence strategy to mitigate the downregulation of global cap-dependent translation in cells resultant from infection-induced ER stress and/or activation of cellular antiviral responses [40,41,43,44]. Conversely, cap-independent translation could allow uncapped genomes that are packaged into virions to establish a RO upon infection, at which point progeny positive-strand genomic RNAs would be capped via the viral machinery [39–41,43]. In either case, the inefficiency of this approach implies that the function of this secondary noncanonical translation mechanism is more of a complement to primary cap-dependent translation, rather than a direct substitute.

## What mediates the switch from translation to replication in the flavivirus life cycle?

The switch from translation to replication is thought to be driven by ribosome exclusion and RO biogenesis [45]. As positive-sense RNA viruses, flaviviral genomic RNAs are ribosome ready and can be translated immediately upon genome uncoating into the cytoplasm. However, once enough viral proteins have accumulated, flaviviruses are met with a dilemma – ribosomes travel in the 5´ to 3´ direction, while the viral NS5 RdRp travels in the 3´ to 5´ direction. Thus, translation and viral RNA replication cannot occur simultaneously on a single viral genomic RNA molecule. As such, there must be a mechanism to switch off translation for viral RNA replication to occur [45,46]. Presumably, the viral genomic RNA must also be efficiently condensed into a RO to establish viral RNA replication [17,45]. RO biogenesis likely occurs somewhat concurrently with ribosome exclusion from the viral genomic RNA and may contribute to establishing and/or maintaining translational inhibition. In this section, we discuss how viral genome cyclization and SLA interactions with the viral NS5 protein facilitate translational shutoff. We also discuss recent data that indicate that viral protein accumulation and genome compaction may be sufficient to establish ROs, albeit at low efficiency, even in the absence of an active mechanism(s) for ribosome exclusion.

### Genome cyclization and the recruitment of NS5 to SLA contribute to translational shut-off

The importance of flaviviral genome cyclization as a precursor to viral RNA replication is well-established and has been the topic of several recent reviews [17,47]. In brief, genome cyclization involves long-range interactions between RNA elements at the 5´ and 3´ ends of the viral genome, including the 5´-3´UAR, DAR and CS elements, which are mutually exclusive with local RNA structures in the linear genome conformation (Figure 1 and Table 1). For example, in DENV, the highly stable DB pseudoknots in the 3´ UTR are only formed in the linear RNA conformation, implying that there must be a large energetic barrier for RNA rearrangement into the replication-competent circularized form of the genome [48]. However, a precise equilibrium between linear and circular viral RNA seems to be essential for infection as stabilization of either RNA conformation is deleterious to viral RNA accumulation [26,49].

Recent advances indicate that genome cyclization may also be important for switching off translation. Interestingly, genome cyclization was shown to impair 48S ribosomal complex formation on the correct start codon of ZIKV and DENV genomes, inhibiting translation initiation at this site, and resulting in clearance of ribosomes from the viral genomic RNA [35]. This finding suggests that the process of genome cyclization itself promotes translational inhibition and the switch between translation and replication in the flaviviral life cycle. Notably, this interplay between local and long-range structures to switch between these two stages of the viral life cycle provides another clear example of how RNA structures increase the functional capacity of the flaviviral genome.

In addition to the genome cyclization elements, interactions between the viral NS5 protein and the SLA structure at the 5´ terminus of the viral genomic RNA have also been implicated in translational shut-off (Table 1). Similar to genome cyclization, the NS5 protein is able to inhibit translation initiation of ZIKV and DENV genomic RNAs both *in vitro* and in cell culture, in a SLA-dependent manner [34]. Interestingly, the interaction between NS5 and SLA appears to be further stabilized by the proximal SLB structure, suggesting that NS5-SLA interactions may precede genome cyclization in the flavivirus life cycle [19]. While the precise mechanism of NS5-mediated inhibition of translation initiation remains to be deciphered, it is conceivable that NS5 binding may preclude recruitment of translation initiation factors to the 5´ cap. Additionally, NS5-SLA interactions likely induce RNA rearrangements that either promote or involve genome cyclization, perhaps via dimerization of NS5 proteins bound concurrently to SLA at the 5´ terminus and the 3´SL at the 3´ terminus of the viral genome [35,50,51]. Nevertheless, whether binding of NS5 to SLA precedes or is concurrent with genome cyclization, these processes likely occur during pauses in translation of the viral genomic RNA following translational bursts, and together allow the viral RNA to be cleared of ribosomes and subsequently condensed into a RO [52].

### Viral protein accumulation and genome compaction may be sufficient for RO biogenesis even in the absence of active translational inhibition

While translational inhibition is an important element in the switch from translation to replication, RO biogenesis is also likely a central part of this process. Intriguingly, the recent development of a replication-independent plasmid-induced replication organelle biogenesis (pIRO) system revealed that it is possible for both DENV and ZIKV genomic RNAs to form ROs, even in the absence of the 5´ UTR and 5´ cyclization elements, albeit very inefficiently [53]. These results suggest that viral protein accumulation, viral protein-RNA, and RNA-RNA interactions may be sufficient for facilitating RO biogenesis even in the absence of an active translational inhibition mechanism. Viral protein-RNA and RNA-RNA interactions may also aid in the necessary compaction of the flavivirus genome into the RO, perhaps through promoting phase separation or biomolecular condensate formation [45]. This latter process has been implicated in ribosome exclusion, and phase separation is also hypothesized to underlie viral RO biogenesis in positive-sense, negative-sense, and double-stranded RNA viruses [45]. Nevertheless, while the pIRO system indicates that the flaviviral RNA is capable of being condensed into a RO in the absence of the 5´ UTR and genome cyclization, this appears to be a rare event in a system where viral RNAs are produced constitutively [53]. As such, during an infection where a single genome needs to be capable of establishing an infection, NS5-SLA interactions and genome cyclization may help facilitate the switch from translation to replication for *efficient* RO biogenesis.

### Long-range RNA-RNA interactions and cellular RNA-binding proteins may facilitate genome compaction for RO biogenesis

While it is largely unclear at present what specific RNA-RNA and protein-RNA interactions underlie RO biogenesis in flaviviruses, it is likely that some of the same long-range RNA-RNA interactions that are necessary for nucleocapsid formation also promote genome compaction into ROs [30–33]. Furthermore, cellular RNA-binding proteins (RBPs) are also likely to contribute to viral RNA compaction during RO biogenesis. For example, insulin-like growth factor 2 mRNA-binding protein 2 (IGF2BP2) can interact with ZIKV NS5 and both the viral 5´ and 3´ UTRs – an interaction that has been demonstrated to be critical for efficient viral RO biogenesis [54]. However, DENV and WNV do not appear to have an IGF2BP2 dependency, implying that the specific cellular RBPs used to promote RO biogenesis may vary between flaviviruses [54]. Additionally, as the viral genomic RNA must be accessible to NS5 to undergo RNA synthesis, it is likely that the degree of genome compaction in the RO is also important for efficient viral RNA replication. In line with this, cellular Ras GTPase-activating protein-binding protein 1 (G3BP1)-promoted genome compaction of non-translating ZIKV and WNV RNAs has an antiviral effect, impairing proper RO biogenesis [55]. However, the eukaryotic initiation factor 4A (eIF4A) helicase activity counteracts G3BP1-mediated compaction, implying that unwinding of the viral RNA could help prevent over-compaction of the viral RNA during RO biogenesis [55].

Taken together, recent studies have made it clear that a myriad of RNA sequences and structures are important in directing the switch from translation to replication in the flavivirus life cycle, by regulating both translational shutoff and facilitating RO biogenesis.

## How do RNA structures direct viral RNA replication?

Flavivirus RNA replication is a two-step process that involves: 1) negative-strand RNA synthesis, which uses the single-stranded positive-sense genomic RNA as a template and results in a double-stranded (ds) RNA replicative intermediate; and subsequently, 2) positive-strand RNA synthesis, using the dsRNA replicative intermediate as a template. Notably, viral RNA replication is a tightly regulated process, with positive-strand genomic RNAs outnumbering negative-strands on the order of approximately 50:1 during infection [56]. Genome cyclization is implicated in loading of the NS5 RdRp on the 3´ terminus of the positive-strand genomic RNA for negative-strand RNA synthesis. Yet, it remains unclear how the negative-strand is chosen as the template for subsequent positive-strand RNA synthesis, given the starting point is the dsRNA replicative intermediate. For example, the NS5 RdRp has specificity for a 3´-UC dinucleotide, which is present at both the 3´ terminus of the positive- and negative-strands [57]. Moreover, the NS3 helicase requires a 3´ single-stranded overhang to unwind dsRNA; meaning that both NS3 and NS5 would presumably have similar affinity for either end of the dsRNA replicative intermediate. This lack of specificity from the viral proteins indicates that the viral RNAs themselves likely provide the direction necessary for the coupled processes of negative- and positive-strand RNA synthesis, and subsequent capping of newly synthesized positive-sense genomic RNAs. Notably, while SLA is already known to play a crucial role in RNA replication, recent advances regarding the tertiary structure of SLA provide new insights into how the NS5-SLA interaction could provide specificity to the genome replication process. In this section, we highlight new observations in NS5-SLA binding models, discuss how NS5-SLA interactions promote negative-strand RNA synthesis, and provide several models for the initiation and progression of negative-strand RNA synthesis. We then discuss positive-strand RNA synthesis from the dsRNA replicative intermediate, a putative positive-strand promoter, and how NS5-SLA interactions facilitate capping of newly-synthesized genomic positive-sense RNAs.

### Recent advances in NS5-SLA interaction models

**Figure 2.**
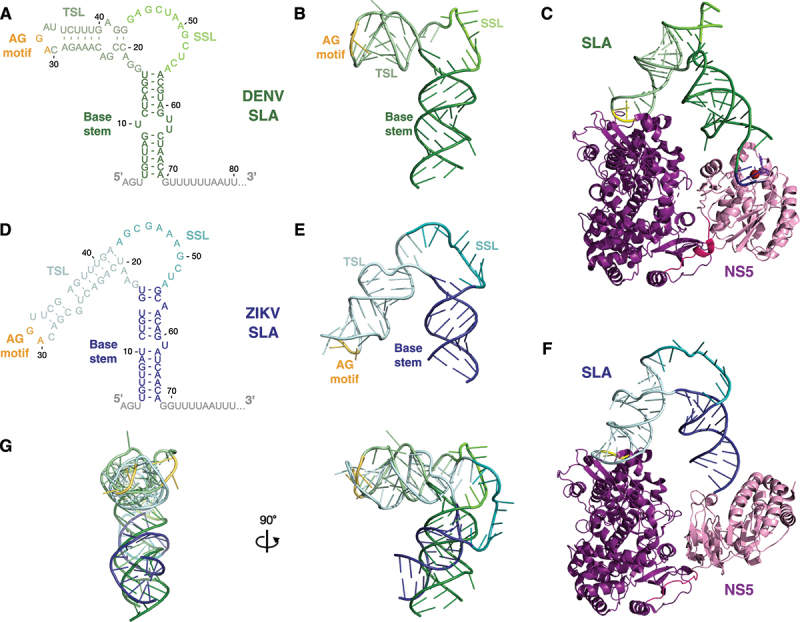
The ‘L/V’ conformations of DENV and ZIKV SLA.

While the interaction between SLA and NS5 remains one of the best characterized interactions between the flavivirus RNA genome and a viral protein, recent advances have challenged the canonical view of the SLA structure. Traditionally, SLA was thought to take on a ‘Y’ shaped conformation, characterized by a base stem, side stem-loop (SSL) and a top stem-loop (TSL) (Figures 1 and 2) [14,58,59]. However, recent crystallographic and cryo-EM-based structural studies suggest that SLA may at least sample an alternative ‘L’ or ‘V’ shaped structure upon NS5 binding (Figure 2) [14,15,60]. In these models, the base stem and TSL are present, but the SSL bases remain unpaired in the central bulge. When interacting with NS5, the base stem and TSL of the ‘L’ or ‘V’ shaped SLA bridge the NS5 MTase and RdRp domains, respectively [14,15]. Kinetic analyses indicate that binding of NS5 to SLA is a 3-step process in which the RdRp first binds to the TSL of SLA, followed by binding of the MTase to the base stem, and finally a rearrangement that stabilizes these interactions [61]. At present, it is unclear whether flavivirus SLAs exist only in the ‘L’ or ‘V’ conformation, or if SLA samples both the ‘Y’ and ‘L/V’ (or other) conformations at various points in the viral life cycle.

A–C) 2D, 3D and NS5-bound conformations of the DENV ‘L’-shaped SLA structure. The base stem, SSL, and TSL of SLA are coloured in dark green, chartreuse, and pale green, respectively. The AG motif of the TSL is indicated in yellow. The structures of SLA and SLA-NS5 of DENV were generated using PDBs:7LYF and 8GZ9, respectively. In C), the DENV NS5 RdRp and MTase domains are coloured in magenta and pink, respectively; with the Mg^2+^ ion coordinating addition of the viral cap to the 5´ terminus indicated in red, while the SAM donor is indicated in light purple. The 5´ terminus of the DENV genome (nt 1-3) is depicted in dark blue.D–F) 2D, 3D and modelled NS5-bound conformations of the ZIKV ‘V’-shaped SLA structure. The base stem, SSL, and TL of SLA are coloured in dark blue, teal, and pale blue, respectively. The AG motif of the TSL is indicated in yellow. The structure of ZIKV SLA was generated using PDB:7LYG and the latter SLA-NS5 model was generated by aligning a ZIKV NS5 monomer from PDB:5M2X and the ZIKV SLA structure (PDB:7LYG; nt 4-69) with their counterparts in the DENV SLA-NS5 structure (PDB:8GZP). Notably, as the ZIKV SLA structure was determined via appending the BSL to a tRNA, we have less confidence in the modeled interactions between SLA and the MT domain than those between SLA and the RdRp domain. In F), the ZIKV NS5 RdRp and MTase domains are coloured in magenta and pink, respectively. G) Structural alignment of SLA structures from DENV (PDB:7LYF) and ZIKV (PDB:7LYG).

These new models for SLA and NS5 binding are largely consistent with known requirements for this interaction. Beyond the overall structural architecture, foot-printing and mutational studies have consistently demonstrated that NS5 binding to SLA also depends on a conserved ‘AG’ motif in the unpaired loop of the TSL that is maintained in all conformations [14,27,51,58,62]. Despite the different angle of the TSL relative to the base of SLA in the ‘L’ and ‘V’ conformations, the AG motif in the unpaired loop of the TSL sits on the same face of the structure, implying that they both interact with their respective NS5 proteins along the same surface (Figures 2b-c & 2e-f) [14]. Furthermore, when the structures of ZIKV and DENV SLA are aligned, the TSL and base stems both largely occupy the same physical space due to different lengths of the helices in ZIKV relative to DENV Figure 2(g) [14]. Thus, ZIKV and DENV SLAs likely bridge the NS5 RdRp and MTase domains in a similar manner Figure 2(c,f). This spatial conservation of NS5 binding interfaces across species provides an explanation for the observation that the NS5s of different flaviviruses are generally interchangeable with respect to SLA binding and that SLA chimeric flaviviruses are replication competent [63,64]. In addition to the TSL ‘AG’ motif, the SSL sequence has also been demonstrated to be important for NS5 binding and viral RNA replication, but it remains unclear whether the sequence, structure, or both, mediate this effect [14,27]. In the ‘L/V’ conformations, potential SLA dimerization via kissing loop interactions of the SSL have been suggested in both DENV and ZIKV SLAs [14]. However, the significance of this potential interaction in flaviviruses remains unclear (*see* **Box 3**) [60]. Interestingly, while the predicted SSL is relatively short (2–3 bp) in the mosquito-borne flaviviruses, it is predicted to be significantly longer (~20 bp) in the tick-borne flaviviruses [58]. It is not yet clear what implication this would have on the ability of SLA to arrange into the putative ‘L/V’ conformation and interact with NS5. Nonetheless, these advances in our understanding of the tertiary structure of SLA and its interactions with NS5 have implications for both negative- and positive-strand RNA synthesis, as well as capping, which will be discussed in detail in the following subsections.

**Table:** 

**Box 3. Is multimerization of flavivirus genomes important for flavivirus infection?** Interestingly, approaches to study flavivirus genome cyclization cannot actually discriminate between *cis-* and *trans*-interactions; and, as such, it has been hypothesized that these elements may also be involved in genome dimerization or multimerization [65]. Thus, rather than genome cyclization (in *cis*), this hypothesis proposes that the effects of the cyclization elements could be due to formation of an antiparallel homoduplex dimer between two genomes or head-to-tail concatemers of multiple flavivirus genomes (in *trans*) [65]. In line with this hypothesis, others have proposed that in the SLA “L/V” conformations of DENV and ZIKV, dimerization may occur via a kissing loop interaction of the SSLs, which was observed at high RNA concentrations *in vitro* [14]. Disruption of the proposed complementary region, which comprises most of the SSL, also impairs viral replication in DENV, but is rescued when swapped with the corresponding region of ZIKV SLA [14]. However, it is not clear from these experiments whether this effect is due to genome dimerization or simply the importance of these nucleotides in interactions with NS5 [27]. Moreover, it remains unclear if this kissing loop interaction can occur in the tick-borne flaviviruses, which have a much longer SSL [58]. Regardless, it seems clear that one or a few viral genomes is sufficient to establish an infection, suggesting that genome dimer/multimerization is unlikely to be an absolute requirement at least at the early stages of the viral life cycle. Nonetheless, it is possible that genome multimerization may occur *in vivo* at later stages in the infection cycle, when high local concentrations of viral genomes are produced [65]. Given that sfRNAs, which contain the 3´ cyclization elements, are also produced during infection, to what extent these can interact with full-length genomic RNAs and modulate the infection process is also an open question [25]. Thus, while genome cyclization (in *cis*) is important for the establishment of an infection, more research will be needed to establish whether genome dimer/multimerization and/or interactions between genomes and sfRNAs (in *trans*) have a role in modulating the course of flavivirus infection.

### NS5 interactions with SLA and the 3´SL promote negative-strand RNA synthesis

**Figure 3.**
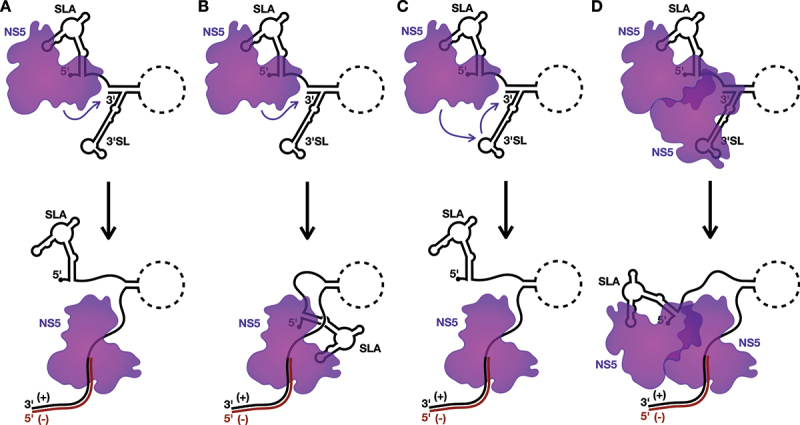
Models for the initiation of negative-strand RNA synthesis.

Recruitment of NS5 by SLA has repeatedly been demonstrated to be critical for replication of the viral genome, but precisely how NS5 localization to SLA at the 5´ end of the viral genome facilitates the initiation of negative-strand RNA synthesis at the 3´ terminus of the positive-sense genomic RNA remains unclear (Table 1). Based on recent data, we envision several models for the initiation of negative-strand RNA synthesis, two of which are SLA-centric, while the latter two additionally rely on interactions with the 3´SL (Figure 3).

A) In model 1, NS5 bound to SLA initiates negative-strand RNA synthesis directly on the adjacent 3´ terminus of the positive-strand. SLA is released during the elongation phase of RNA synthesis. B) In model 2, the NS5 bound to SLA initiates negative-strand RNA synthesis directly on the adjacent 3´ terminus, but remains bound to SLA during elongation, only releasing SLA when it is itself unwound for completion of the dsRNA replicative intermediate. C) In model 3, the NS5 bound to SLA is transferred to the 3´SL due to the higher affinity of NS5 for the 3´SL following genome cyclization-induced unwinding of SLB. NS5 then initiates negative-strand RNA synthesis directly on the 3´ terminus of the positive-strand. D) In model 4, the NS5 bound to SLA interacts with a second NS5 bound to the 3´SL, allowing the latter NS5 to initiate negative-strand RNA synthesis off the 3´ terminus of the positive-strand. The 5´ terminus of the genome remains associated with the SLA-bound NS5, only releasing SLA when it is itself unwound for completion of the dsRNA replicative intermediate.

As a consequence of the ‘L/V’ conformation of SLA, the bound NS5 appears to sit below the SLA structure, where it would be proximal to the 3´ terminus as a consequence of genome cyclization [14,15,60]. As such, in the first model, NS5 could initiate negative-strand RNA synthesis directly off the partially unwound 3´ terminus of the positive-strand (Figure 3A). However, in this first model, transfer of the NS5 from SLA to the 3´ terminus of the positive-strand RNA could potentially create an opening for reinitiation of translation at the now ‘free’ 5´ terminus of the viral genome [34,35]. Thus, in the second model, NS5 remains bound to SLA as it synthesizes the negative-strand, only releasing SLA when it needs to be unwound for completion of the dsRNA replicative intermediate (Figure 3B). Importantly, this continued engagement with the 5´ terminus during RNA synthesis from the 3´ terminus is not unprecedented, as several viral RdRps, including those of coronaviruses and influenza viruses, have been proposed to either directly or indirectly interact with the 5´ terminus of the template RNA as they synthesize the complementary strand [66–69]. Since SLA remains bound to NS5 during the elongation phase of viral RNA synthesis in this second model, sequestration of the 5´ terminus could also help prevent reinitation of translation, which may be advantageous if compaction of the viral RNA into a RO is occurring concurrently with the initiation of negative-strand RNA synthesis.

However, these SLA-centric models do not directly account for the role of NS5–3´SL interactions in viral RNA replication [50,51,70,71]. Notably, the 3´SL also contains a conserved ‘AG’ dinucleotide in its TSL that has been shown to be critical for viral RNA replication and NS5 recruitment, and could facilitate NS5–3´SL binding similarly to NS5-SLA interactions [14,15,27,51,58,60,62,70,71]. To account for this interaction, in the third model for the initiation of negative-strand RNA synthesis, the NS5 bound to SLA is transferred to the 3´SL due to the higher affinity of NS5 for the 3´SL over SLA following the cyclization-induced unwinding of SLB [19]. Subsequently, the transferred NS5 could then initiate at the 3´ terminus to synthesize the negative-strand replicative intermediate (Figure 3C) [72]. However, this model has the same potential translation reinitiation drawback as the first model, which leads us to the final model, whereby the NS5 bound to SLA does not directly synthesize the negative-strand, but rather interacts with a second NS5 bound to the 3´SL, and facilitates rearrangement to allow this latter NS5 molecule to initiate negative-strand RNA synthesis in the context of an NS5 dimer (Figure 3d). Similarly to the second model, the 5´ terminus would thus remain associated with the SLA-bound NS5 during negative-strand RNA synthesis until this region requires unwinding for completion of the dsRNA replicative intermediate. While it is well accepted that flaviviral NS5 can dimerize (or even form higher-ordered oligomers), the orientation of a putative NS5 dimer in the initiation of negative-strand RNA synthesis remains unclear, as several NS5 dimer/oligomer orientations have been observed in crystal structures [73–75]. While more experimentation will be needed to provide definitive support for any one of these four models, it seems clear that *cis*-acting RNA elements, including SLA and perhaps also 3´SL, as well as the genome cyclization sequences, play key roles in directing the initiation of negative-strand RNA synthesis in the flavivirus life cycle.

### SLA may also have an active role in positive-strand RNA synthesis

**Figure 4.**
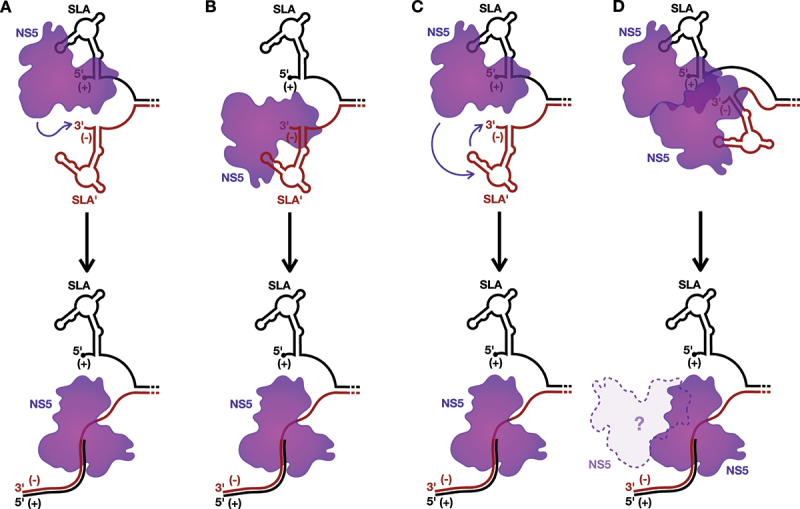
Models for the initiation of positive-strand RNA synthesis.

Compared with negative-strand RNA synthesis, the requirements for positive-strand RNA synthesis are more poorly defined, although it seems increasingly likely that SLA also plays a central role in this process (Figure 4 and Table 1). The major difference between negative- and positive-strand RNA synthesis relates to the nature of the template RNA, which is single-stranded positive-sense genomic RNA and the dsRNA replicative intermediate, respectively. Although each end of the dsRNA replicative intermediate is biochemically similar and possesses all the features needed for the core replicase to act, there is a clear preference for the initiation of positive-strand RNA synthesis relative to negative-strand RNA synthesis [56,57,76]. As discussed previously, since the specificity needed to maintain this preference does not come from the viral proteins involved, it is likely that features of the viral RNAs themselves are responsible for guiding this process. A recent study that used ribozymes to explore internal RNA folding in negative-strand replicative intermediates suggests that regions of the negative-strand in YFV, the related hepatitis C virus (HCV), and other positive-sense RNA viruses can be locally structured during viral replication, albeit at low frequency [77]. Thus, perhaps in combination with terminal ‘breathing’ or a yet undiscovered active unwinding mechanism, SLA may be able to spontaneously reform at the 5´ terminus of the positive-strand in the dsRNA replicative intermediate [78].

A) In model 1, NS5 bound to SLA initiates positive-strand RNA synthesis directly on the adjacent 3´ terminus of the negative-strand within the dsRNA replicative intermediate. B) In model 2, NS5 is recruited directly to SLA´ to initiate positive-strand RNA synthesis directly on the 3´ terminus of the negative strand. C) In model 3, the NS5 bound to SLA is transferred to SLA´ and initiates positive-strand RNA synthesis directly on the 3´ terminus of the negative-strand within the dsRNA replicative intermediate. D) In model 4, the NS5 bound to SLA interacts with a second NS5 bound to SLA´, allowing the latter NS5 to initiate positive-strand RNA synthesis off the 3´ terminus of the negative-strand within the dsRNA replicative intermediate. It is unclear whether the NS5 originally bound to SLA would dissociate from or remain bound to the elongating NS5 carrying out positive-strand RNA synthesis. In all cases, the positive-strand is displaced from the dsRNA replicative intermediate during synthesis of the new positive-strand.

As such, SLA could also play a role in directing the initiation of positive-strand RNA synthesis, in parallel to the models discussed above for the initiation of negative-strand RNA synthesis (Figures 3 & 4). For example, binding of NS5 to the re-folded SLA could stabilize the 5´ terminus of the positive-strand, resulting in a single-stranded 3´ overhang on the negative-strand that is necessary for NS3 to initiate unwinding of the dsRNA intermediate [76]. Similarly to the first model for negative-strand RNA synthesis (Figure 3A), it is possible that the NS5 recruited to the reformed SLA could then directly interact with the 3´ terminus of the negative-strand to initiate positive-strand RNA synthesis from the dsRNA template (Figure 4A). Alternatively, akin to the role of 3´SL in the models for negative-strand RNA synthesis (Figure 3C,d), it is possible that the terminal structure at the 3´ end of the negative-strand, termed SLA´ herein, serves as the promoter for positive-strand RNA synthesis (Figure 4B-D). Interestingly, despite the presence of a couple alternative G:U wobble base pairs, SLA´ is predicted to essentially form a mirror image of SLA, with a base stem, SSL, and a conserved unpaired ‘AG motif’ in its TSL [79]. This mirroring is somewhat unexpected, as in the related HCV, the structure of the 5´ terminus of the positive-strand and the 3´ terminus of the negative-strand possess completely independent architectures due to G-U wobble base pairing, and the latter has been demonstrated to serve as the promoter for positive-strand RNA synthesis [80–82]. Furthermore, in support of a role for flaviviral SLA´ in positive-strand RNA synthesis, *in vitro* binding studies with JEV show that NS5 has a greater affinity for the 3´ terminus of the negative-strand than the 3´ terminus of the positive-strand [83]. Moreover, in ZIKV, the terminal 105 nucleotides at the 3´ terminus of the negative-strand is important for efficient regulation of positive-strand RNA synthesis *in vitro*, a region which includes SLA´ [84]. As such, it is possible that NS5 is directly recruited to SLA´ (Figure 4B), or transferred from SLA to SLA´ (Figure 4C), to initiate positive-strand RNA synthesis. Alternatively, the NS5 bound to SLA could interact with, or even aid in the recruitment of, a second NS5 bound to SLA´, to promote initiation of positive-strand RNA synthesis (Figure 4D). However, in contrast to the fourth model for negative-strand RNA synthesis (Figure 3D), it seems less clear during the elongation stage whether the NS5 bound to SLA would be displaced or remain bound to the NS5 carrying out positive-strand RNA synthesis, since the displaced positive-strand needs to exit the RO to maintain the largely uniform size of the flaviviral RO (Figure 4D) [4,5]. Beyond these models, it is also possible that the SLA´ structure may simply serve to promote positive-strand RNA synthesis by reinforcing strand separation at the terminus of the dsRNA replicative intermediate. Regardless, both SLA and/or SLA´ would presumably need to reform for each subsequent round of positive-strand RNA synthesis, as this process results in regeneration of the dsRNA intermediate. Nevertheless, while the mechanism of initiating positive-strand RNA synthesis remains largely a black box, it is likely that SLA, and possibly also SLA´, help direct the process.

### NS5-SLA interactions provide specificity for genome capping

In addition to its roles in viral RNA replication, SLA has also been implicated in the capping of newly-synthesized viral genomes (Table 1). While the RTPase activity of NS3 is responsible for reducing the 5´ triphosphate to a monophosphate moiety, the latter steps of the capping reaction are catalysed by NS5’s MTase activity, which adds a guanyl cap and then methylates at the *N*-7 and 2´-O positions of the 5´ cap. In both DENV and WNV, 2´-O-methylation (2´-OMe) of the A1 position of the genome is RNA structure-independent, and only dependent on a G at position 2, which stacks with the A1 base and aids in coordinating the Mg^2+^ ion bound to NS5 [85,86]. Together with the requirement of the NS5 RdRp to initiate RNA synthesis on a 3´-UC dinucleotide, these requirements for 2´-OMe contribute to the strict conservation of an AG dinucleotide at the 5´ terminus of the RNA [57]. In contrast to 2´-OMe, efficient *N*-7 methylation in WNV appears to be SLA-dependent, with the interaction between the base stem and NS5 being of particular importance [85]. NS5 binding to the ‘L/V’ conformation of SLA in DENV and ZIKV is consistent with a SLA-mediated capping model, with the capped 5´-tetranucleotide (5´-GpppAGUU) well accommodated in the catalytic pocket of the NS5 MTase domain (Figures 2C & 2F) [14,15]. While the addition of the G cap itself has not been studied in the context of SLA, the positioning of the 5´ terminus in the MTase active site implies that this step is also likely to be SLA-mediated. Thus, beyond the importance of SLA-NS5 interactions in providing the specificity for regulating negative- and positive-strand RNA synthesis, these new interaction models also reveals a role for SLA-NS5 interactions in viral RNA capping.

## What features of the genomic RNA provide specificity for genome packaging?

To date, virion assembly remains structurally and biochemically one of the least defined stages of the flavivirus life cycle [87,88]. In brief, the nascent viral genomic RNA must be localized to the assembly site (often located in close proximity to the RO pore), condensed, and encapsidated as it buds into the ER lumen, gaining a viral envelope studded with the viral pre-membrane and envelope proteins [88]. However, at least *in vitro*, the capsid protein does not appear to have any specificity for the viral genomic RNA, raising the question as to how viral genomes are specifically packaged into assembling viral particles [89]. Recent data has indicated that the viral NS2A protein may provide specificity for genome packaging, through specific interactions with the viral 3´ UTR [28,29]. In this section, we will discuss the RNA structures and sequences of the putative viral packaging signal, as well as long-range RNA-RNA interactions that likely facilitate compaction of the viral genome into the assembling virion.

### NS2A–3´ UTR interactions facilitate packaging of flavivirus genomic RNAs

Having no clearly defined ‘packaging signal’ within the flaviviral RNA, the mechanism(s) surrounding specificity in genome packaging have remained elusive [17]. However, interactions between NS2A and the 3´ UTR are necessary for genome packaging in ZIKV, DENV, and Kunjin virus (KUNV), suggesting that NS2A–3´ UTR interactions provide specificity to the virion assembly process (Table 1) [28,29]. However, the 3´ UTR structures bound by NS2A appear to differ across these flaviviruses. Based on competitive pull-down experiments, ZIKV NS2A binds to SL-II specifically, while the last 285 nucleotides of the 3´ UTR (corresponding to DBI, DBII, sHP and the 3´SL) are needed for successful interaction with DENV NS2A, implying that DENV NS2A interacts with the overall RNA structure in this region rather than an individual structure [28,29]. Taken together, these findings imply that there is no singular conserved RNA structure that acts as the flaviviral packaging signal, but instead flavivirus NS2A proteins may have each divergently evolved to recognize RNA sequences and/or structures in the 3´ UTR as a means of providing specificity to the virion assembly process.

Beyond interacting with the 3´ UTR of the viral genome, NS2A is hypothesized to serve as a central hub at the virion assembly site due to its interactions with the other viral proteins required for assembly (including the capsid, pre-membrane, and envelope complex, as well as NS2B and NS3). The ability of NS2A to facilitate the co-localization of the viral genome and capsid proteins would bring them into close proximity, possibly driving genome condensation and formation of the nucleocapsid [28,29,90]. The NS2A–3´ UTR interaction could thereby circumvent the capsid protein’s apparent lack of specificity for the viral RNA genome, and thus serve as a noncanonical packaging signal for the flavivirus genomic RNA [91].

### Long-range RNA-RNA interactions likely facilitate compaction of the viral RNA into the assembling virion

The structure of the packaged genome has not been elucidated in recent high-resolution cryo-electron microscopy studies of flaviviral virions, but the need for compaction of the viral genomic RNA to package it inside the virion is clear [92]. For instance, the hydrodynamic radius of the DENV2 genome is ~45 nm in the absence of viral and host proteins, while its virion has a radius of ~25 nm, meaning that the viral genome needs to be reduced to <17% of its volume to be successfully packaged [30]. In addition, the need to compact the viral genome is not limited to virion assembly, but is also a requirement for RO biogenesis, as discussed above [45]. To solve this dilemma, the genomes of flaviviruses have evolved to have numerous long-range RNA-RNA interactions (generally greater than 0.5 kb of separation between the interacting elements) (Table 1) [30–33]. In infected cells, only 34% of RNA interactions are classified as long-range, while this number rises to 77% when looking solely at RNA within the virion [33]. Interestingly, there appears to be high plasticity in these interactions across flaviviruses. Rather than utilizing the same long-range RNA-RNA interaction in a particular region of the genome, different flaviviruses have often evolved a comparable interaction close by that promotes genome compaction [30,33]. Even within a single strain, a given RNA element is more likely to have an alternative long-range RNA interaction partner within a packaged virion than within an infected cell, pointing towards an overall structural heterogeneity to compact the genome into the assembling virion [32]. Despite this heterogeneity and plasticity in the RNA-RNA interactions used to condense the genome, the overall degree of these interactions appears to be tightly balanced, as disruption of several individual long-range RNA-RNA interactions in DENV was sufficient to impair viral fitness and increase the genome volume by ~30–50% [30]. Interestingly, while the DENV capsid protein does not form specific interactions with the genomic RNA, it has been shown to preferentially bind to regions of the genome involved in these long-range RNA-RNA interactions within the virion [31]. Thus, genome compaction could also be assisted during virion assembly by capsid’s RNA chaperone activity. This relationship between compaction and encapsidation would be mutually synergistic, with long-range RNA-RNA interactions in the genome facilitating capsid binding, while capsid in turn aids in their stabilization and thus promotes long-range RNA-RNA interactions [89]. In addition to capsid, cellular RBPs also contribute to the stability and remodelling of these long-range RNA-RNA interactions (*see* **Box 4**). Overall, the formation of long-range RNA-RNA interactions is a highly plastic, but critical step in genome compaction and encapsidation.

**Table:** 

**Box 4. How do cellular RBPs participate in genome compaction?** To accomplish the ambitious task of compacting the viral genomic RNA into the assembling virion, it seems likely that cellular RBPs must lend a helping hand. For example, the DEAD box (DDX) helicase, DDX56, relocalizes from the nucleus to the site of WNV assembly during infection, and its helicase activity is important for efficient virion production [93,94]. In general, DDX proteins are known to regulate RNA condensation, as they remodel RNA-RNA and protein-RNA interactions through their helicase activity [95]. As such, it is tempting to speculate that DDX56 promotes genome compaction during WNV assembly via remodelling RNA-RNA and protein-RNA interactions with the viral genomic RNA. Interestingly, several DDX family proteins are also implicated in flaviviral RNA replication, suggesting that genome compaction for RO biogenesis may also require DDX helicase activity [96]. Additionally, cellular RBPs may also promote genome compaction by providing additional protein-RNA interactions to condense the viral RNA. For example, the YBX-1 protein has been shown to interact with the 3´SL, which inhibits viral translation and has a detrimental impact on DENV infection; but this may be mitigated by the generation of sfRNAs, which are known to sequester cellular RBPs [97,98]. On the other hand, YBX-1 is also known to be required for proper virion assembly, through interactions with the viral genomic RNA as well as the DENV capsid and envelope proteins [99,100]. However, it is unclear if the interaction with the viral RNA during virion assembly is solely through the established interaction with the 3´SL, or if YBX-1 interactions provide multivalency for genome compaction by binding to additional sites across the DENV genome [98]. Nonetheless, these examples suggest that cellular RBPs also interact with flaviviral RNAs and help modulate genome compaction during both RO biogenesis and virion assembly.

Taken together, both specific interactions between the NS2A protein and the 3´ UTR of the viral genomic RNA (that likely serves as the viral packaging signal), as well as more loosely defined long-range RNA-RNA interactions across the viral genomic RNA (which facilitate genome compaction and encapsidation), help drive genome packaging in the flavivirus life cycle.

## Concluding remarks

In summary, three major themes are starting to emerge regarding the general roles played by viral RNA structure in the flavivirus life cycle, with parallels in a diverse range of RNA viruses. These themes include providing a layer of specificity across the viral life cycle, increasing the functional capacity of the genome, and providing a mechanism to support genome compaction.

The first of these themes is that RNA structure provides specificity to a tightly regulated system in which the viral proteins generally act non-specifically. During translation, the viral genomic RNA provides specificity via the cHP, which ensures proper start codon selection [22]. Interestingly, alphaviruses utilize a similar mechanism, whereby a stable downstream stem-loop on the subgenomic mRNA is hypothesized to form an RNA trap and stall ribosomal scanning at the start codon for production of the viral structural proteins [20,101,102]. Since stable stem-loop structures shortly downstream of the start codon lower the requirement for optimal start codon context in eukaryotic translation, it is not surprising that this mechanism of start codon selection extends broadly across RNA viruses [103]. Additionally, during RNA replication, SLA provides specificity to negative- and positive-strand RNA synthesis, and the capping reaction through its interactions with NS5, the viral RdRp and MTase. Similarly, the poliovirus 5´ cloverleaf structure is also known to recruit the viral RdRp and promote both negative- and positive-strand RNA synthesis [104]. Furthermore, while the mechanism of positive-strand RNA synthesis initiation is largely unknown in flaviviruses, RNA structures at the 3´ terminus of the negative-strand of the closely related HCV also serve as the promoter for initiating positive-strand RNA synthesis [80–82]. Beyond positive-sense RNA viruses, the 5´ and 3´ termini of the influenza virus genome segments (a multi-segmented negative-sense RNA virus) form a corkscrew-like structure that recruits the viral RdRp [105]. Thus, the concept of 5´ and 3´ terminal RNA structures directing viral RNA synthesis is also likely to apply broadly across RNA viruses. Finally, interactions between NS2A and structures in the 3´ UTR of flaviviruses provide specificity for genome packaging. Analogous to differences in the packaging signals in the related ZIKV and DENV, distinct betacoronaviruses have vastly different structural requirements in their packaging signals, ranging from a 95-nt structure in mouse hepatitis virus to a 1.4 kb region in severe acute respiratory syndrome coronavirus 2 (SARS-CoV-2), further illustrating the diversity of packaging signals even among highly related viruses [106–108]. Conversely, selective genome packaging of each of the eight influenza virus genome segments relies upon RNA-RNA interactions between the packaging signals of the different genome segments, providing an elegant solution to a complex packaging specificity problem [109]. Taken together, these examples help to demonstrate how viral RNA structure provides specificity, not only in flaviviruses but across RNA viruses more broadly.

The second theme regarding the role of RNA structure in the flavivirus life cycle is to increase the functional capacity of their small RNA genomes, in which the ~11 kb RNA must encode the 10 viral proteins and all the functional sequence/structural information needed to carry out the complex infectious cycle. For example, encoding a cap-independent mechanism for translation initiation provides a layer of redundancy in times of stress (*see* **Box 1**). Possessing such a cap-independent mechanism is incredibly valuable to RNA viruses as infection generally leads to downregulation of cellular cap-dependent translation due to interferon-induced phosphorylation of the eukaryotic initiation factor 2α [40,41,43,44]. As such, it is not surprising that many RNA viruses, including poliovirus and HCV, encode IRES structures that allow them to recruit the ribosome in a cap-independent manner using a fraction of the eukaryotic translation initiation factors [110]. Another example of how RNA structure increases the functional capacity of flavivirus genomes are their mechanisms for translational shutoff. In a parallel manner to NS5-SLA interactions in the flavivirus life cycle, the cloverleaf structure at the 5´ terminus of the poliovirus genome also participates in translational shutoff and the switch from translation to replication [67]. Additionally, like the cyclization elements of flaviviruses, interactions between the poly-C binding protein 2 and the poliovirus cloverleaf structure at the 5´ terminus and poly-A binding protein at the 3´ terminus aid in genome circularization for viral RNA replication [67]. Mechanisms for genome circularization are pervasive across diverse RNA viruses, representing a common approach for increasing the functional capacity of their genomes [66–69]. Interestingly, these genome circularization mechanisms inhibit viral translation in both flaviviruses and picornaviruses [26,67]. This observation may also be true for coronaviruses, where nucleocapsid-mediated circularization promotes negative-strand RNA synthesis, although the mechanism is still unclear [68]. As such, circularization of the genomes of positive-sense RNA viruses may represent a common mechanism for mediating translational shut-off. Beyond the structures covered in this review, there are several well-characterized examples of alternative RNA conformations that aid in increasing the functional capacity of viral RNAs. These include the flaviviral pseudoknots, which promote sfRNA production to evade antiviral responses; the subgenomic mRNA promoter region of togaviruses; and the transcriptional regulatory sequences in coronaviruses that mediate discontinuous transcription for the production of subgenomic mRNAs [24–48–101,102–113]. Thus, it seems clear that RNA secondary and tertiary structures allow RNA viruses to increase the functional capacity of their genomes beyond that of their limited coding capacity.

Finally, the third major theme regarding the role of RNA structure in the viral life cycle is compaction of the viral genome. In all RNA viruses, there are two major stages of the viral life cycle that require viral genome compaction: 1) RO biogenesis, in which the viral RNA is condensed together with viral replication proteins into a replication compartment; and 2) virion assembly, in which the viral genome is condensed into the nucleocapsid and infectious viral particle [30,45]. In flaviviruses, it appears that genome compaction is driven by long-range RNA-RNA interactions across the viral genomic RNA [30–33]. While similar whole-genome analyses have been performed in other RNA viruses, these studies have largely not included information on long-range RNA-RNA interactions [114]. However, a recent study of SARS-CoV-2 genome structure revealed numerous long-range RNA-RNA interactions within the virion, resulting in an unentangled globule conformation of the genome [115]. It is unclear at present whether these long-range RNA-RNA interactions promote compaction of the viral genome, as they do in flaviviruses, or are a consequence of compaction of the viral genome into the assembling virion. Nonetheless, RNA secondary and tertiary structures are likely to play a central role in the process of genome compaction for both RO biogenesis and virion assembly.

Despite recent advances in our understanding of the roles of RNA structure in the flavivirus life cycle, there remain a number of outstanding questions and challenges in elucidating the precise roles of RNA structures during infection. For example, to further interrogate which of the aforementioned models for negative- and positive-strand RNA synthesis best reflects the role of SLA-NS5 interactions, it will be necessary to develop experimental systems that uncouple the potential roles of SLA in the switch from translation to replication, negative- and positive-strand RNA synthesis, and genome capping (Figures 3 and 4). The complexity needed for such a system has thus far limited efforts to further interrogate each individual role of SLA, and the overlaps in sequence and structural requirements for SLA across these roles renders the application of traditional stem disruption and rescue approaches for assessing function very difficult in the context of the viral life cycle. Moving forward, creative approaches that allow the isolation of one or more of these roles, analogous to the pIRO system for analysing requirements for RO biogenesis independently of viral RNA replication, will be needed [4]. While such systems may not provide direct equivalents to infection, they can provide insight into the players involved and will help to refine models for viral translation, replication, and packaging in the flavivirus life cycle.

Lastly, that a single piece of viral genomic RNA could play such myriad roles across the viral life cycle speaks to the highly sophisticated nature of flaviviruses. Moreover, the themes discussed herein, including specificity, functional capacity, and genome compaction extend beyond flaviviruses, with parallel roles described for RNA structures in the life cycles of a diverse array of RNA viruses.

## Data Availability

The authors confirm that the data supporting the findings of this study are available within the article.

## Highlights

- RNA structures provide a layer of specificity across the flavivirus life cycle.
- RNA sequence and structural elements, and their dynamics, increase the functional capacity of flaviviral genomes.
- SLA is a critical element in the switch from translation to replication, negative- and positive-strand RNA synthesis, and capping of newly-synthesized genomic RNAs.
- New models for interactions between SLA and NS5 shed light on potential mechanisms for the initiation of negative- and positive-strand RNA synthesis.
- Long-range RNA-RNA interactions in flavivirus genomes are highly plastic and critical for compaction of the viral genome into replication organelles and infectious viral particles.
